# Solid component proportion is an important predictor of tumor invasiveness in clinical stage T_1_N_0_M_0_ (cT_1_N_0_M_0_) lung adenocarcinoma

**DOI:** 10.1186/s40644-018-0147-7

**Published:** 2018-05-04

**Authors:** Meng Li, Ning Wu, Li Zhang, Wei Sun, Ying Liu, Lv Lv, Jiansong Ren, Dongmei Lin

**Affiliations:** 1 Department of Diagnostic Radiology, National Cancer Center/Cancer Hospital, Chinese Academy of Medical Sciences and Peking Union Medical College, Beijing, China; 2 PET-CT Center, National Cancer Center/Cancer Hospital, Chinese Academy of Medical Sciences and Peking Union Medical College, Beijing, China; 3 Department of Pathology, Beijing Cancer Hospital, Beijing, China; 4 National Office for Cancer Prevention and Control, National Cancer Center/Cancer Hospital, Chinese Academy of Medical Sciences and Peking Union Medical College, Beijing, China

**Keywords:** Lung adenocarcinoma, Neoplasm invasiveness, Computer tomography, Positron-emission tomography, CT number analysis

## Abstract

**Background:**

Preoperative tumor invasiveness in clinical stage T_1_N_0_M_0_ lung adenocarcinoma is critical for optimal surgical procedure. The aim of the present study was to evaluate the relationship between the ground-glass opacity component (GGOc) / solid component (Sc) proportion measured using three-dimensional (3D) computer-quantified computer tomography (CT) number analysis to explore radiographic features for invasiveness prediction in cT_1_N_0_M_0_ lung adenocarcinomas.

**Methods:**

A total of 375 surgically resected cT_1_N_0_M_0_ lung adenocarcinoma patients were included. The relativity between the GGOc/Sc proportion and lepidic growth pattern percentage was assessed using Spearman’s rank analysis. Multiple logistic regression analysis was used to determine independent factors from radiographic features for tumor invasiveness. Prediction probability for tumor invasiveness was analysed using a receiver operating characteristic curve (ROC).

**Results:**

We found that the GGOc proportion was positively correlated with lepidic growth pattern percentage (*r* = 0.67, *P* <  0.01), while the Sc proportion was negatively correlated with it (*r* = − 0.74, *P* <  0.01). Multivariate analysis showed that tumor size and Sc proportion were identified as independent predictors for tumor invasiveness. The area under the ROC curve (AUC) of Sc proportion was 0.875, which was higher than that of tumor size (0.750) (*P* <  0.001), and had no significant difference with that of combination of these two factors (0.884) (*P* = 0.28).

**Conclusions:**

The GGOc/Sc proportion measured using 3D computer-quantified CT number analysis reflects the lepidic growth pattern percentage in tumors, and the Sc proportion may be an important factor for the prediction of tumor invasiveness in cT_1_N_0_M_0_ lung adenocarcinoma.

## Background

Lung adenocarcinoma (ADC) is the most common histologic subtype of lung cancer in most countries, and an upward trend in ADC incidence has been investigated [1, 2]. Many advances have occurred in oncology, molecular biology, pathologic examination, radiology, and surgery of lung ADC. With this background, the International Association for the Study of Lung Cancer, the American Thoracic Society and the European Respiratory Society (IASLC/ATS/ERS) proposed a new classification of lung ADC in 2011 [3, 4], which is closely related to clinical prognosis and has been identified as an independent prognostic factor in the disease-free survival and overall survival of patients [5]. Previous studies have documented that the 5-year disease-free survival of patients with adenocarcinoma in situ (AIS) was 100%, with minimally invasive adenocarcinoma (MIA) near 100% and invasive adenocarcinoma ranging from 40% to 85% [5–7].

The difference in clinical prognosis is an important basis for individual treatment plan. Although the role of limited resection awaits the results of two randomized trials (JCOG 0802 in Japan and CALGB 140503 in North America) [8, 9], previous studies have suggested that sublobar (limited) resection alone without adjuvant therapy is the optimal choice for AIS/MIA because of the satisfactory prognosis, and some of the cases involved multiple primary adenocarcinoma [10, 11]. Invasive adenocarcinoma may need lobectomy and adjuvant therapy according to its pathological subtypes and other clinicopathological factors [3]. Therefore, pre- or intraoperative diagnosis is critical to select an optimal surgical procedure. However, it is difficult for pathologists to decide tumor invasion on preoperative and intraoperative frozen sections [3, 12]. Thus, preoperative radiography is necessary and important to help predict tumor invasiveness and determine the most appropriate surgical procedures.

Three-dimensional (3D) computer-quantified CT number analysis is used to determine the proportion of tissue component by calculating the proportion of one CT number or a range of CT numbers in the lesion [13–15]. We hypothesized that the tissue component proportion in CT images measured using a 3D computer-quantified CT number analysis is associated with the pathological constituents percentage, and this components proportion and other radiographic features can help predict tumor invasiveness. Therefore, the aim of the present study is to evaluate the relationship between the ground-glass opacity component (GGOc)/solid component (Sc) proportion measured using 3D computer-quantified CT number analysis to explore radiographic features for invasiveness prediction in cT_1_N_0_M_0_ lung adenocarcinomas.

## Methods

### Patients

A total of 375 consecutive patients with surgically resected cT_1_N_0_M_0_ lung adenocarcinoma at our hospital between January 2005 and December 2012 were included. The following inclusion criteria were considered for the present study: **(a)** single adenocarcinomas (3 cm or less in diameter at CT image) with no evidence of malignant satellite nodules (previously confirmed with imaging study or lung biopsy) and no hilar or mediastinal lymphadenopathy on imaging study or at mediastinoscopy, **(b)** first treatment with surgery alone, **(c)** either chest high-resolution computer tomography (HRCT) studies or integrated 18 fluorodeoxyglucose positron emission tomography (^18^F-FDG PET)/CT acquired within 1 month for preoperative staging before resection, and **(d)** both pathological sections and clinical data are available for review.

### Image acquisition

The CT images were obtained using an eight-(LightSpeed Ultra, GE Medical Systems), 16-(ProSpeed or Discovery ST, GE Medical Systems) or 64-(LightSpeed VCT, GE Medical Systems or Toshiba Aqulion, TOSHIBA Medical Systems) slice spiral CT scanner. CT images were obtained with 120 kVp, 250 ~ 350 mA, and a reconstruction kernel with standard algorithm. Reconstruction thicknesses were 1.0 or 1.25 mm, and the intervals were 0.8 or 1.0 mm. With enhanced CT examination, 80 ml intravenous contrast was administered at 2.5 mL/s, and images were obtained 25 to 30 s after contrast infusion.

PET/CT images were acquired using an integrated PET/CT device (Discovery ST 16, GE Medical Systems). After confirmation of normal blood glucose levels in the peripheral blood was ensured (≤8 mmol/L), the patients were administered an intravenous injection of ^18^F-FDG at 3.70 ~ 4.44 MBq/kg and subsequently rested for approximately 60 ~ 70 min before undergoing the body scan. The whole body scan was performed from the head to root of the thigh, and the thoracic scan was performed from the super-clavicle to the adrenal gland. The patients who did not undergo chest CT examination within 10 days underwent a breath-hold thoracic spiral CT scan with 120 kV, 205 mA after the PET/CT scan.

### Image interpretation

CT images were retrospectively assessed for visual morphological features. Two radiologists (M.L. and L.Z.), who had 11 and 8 years of experience, respectively, were informed that the involved patients had surgically treated adenocarcinomas but were blinded to histologic subtypes and conducted the analysis of the morphological features using a Carestream GCRIS 2.1 PACS workstation (Carestream Health) in consensus. The morphological features included CT appearance (solid nodules [SN], part solid nodules [PSN] or mGGO [mixed ground-glass opacity], pure ground-glass opacity [pGGO]), tumor size (the longest tumor diameter on the transverse lung window image, where the largest nodule dimension appeared), location (centre or periphery, periphery was defined as within 3 cm of the pleura), contour (smooth, lobular or spiculated), necrosis (necrosis of tumor was defined as low attenuation in tumor), and vacuole sign or cyst/cavity (vacuole sign was defined as a gas-filled space less than 0.5 cm in tumor while cyst/cavity was no less than 0.5 cm) [16, 17].

Two chest radiologists with PET/CT diagnostic experience (N.W. and Y.L.) retrospectively evaluated the integrated PET/CT images. For a semi-quantitative analysis of FDG uptake, regions of interest (ROI) were placed over the tumor site on the hottest trans-axial slice. In some patients, nodular FDG uptake could not be identified on the PET component images. For these patients, a ROI was drawn in a presumed nodular location, considering the CT component images of PET/CT. The maximum standard uptake value (SUV_max_) within the ROI was used as the reference measurement.

### CT number analysis by three-dimensional computerized quantification

**Fig. 1:**
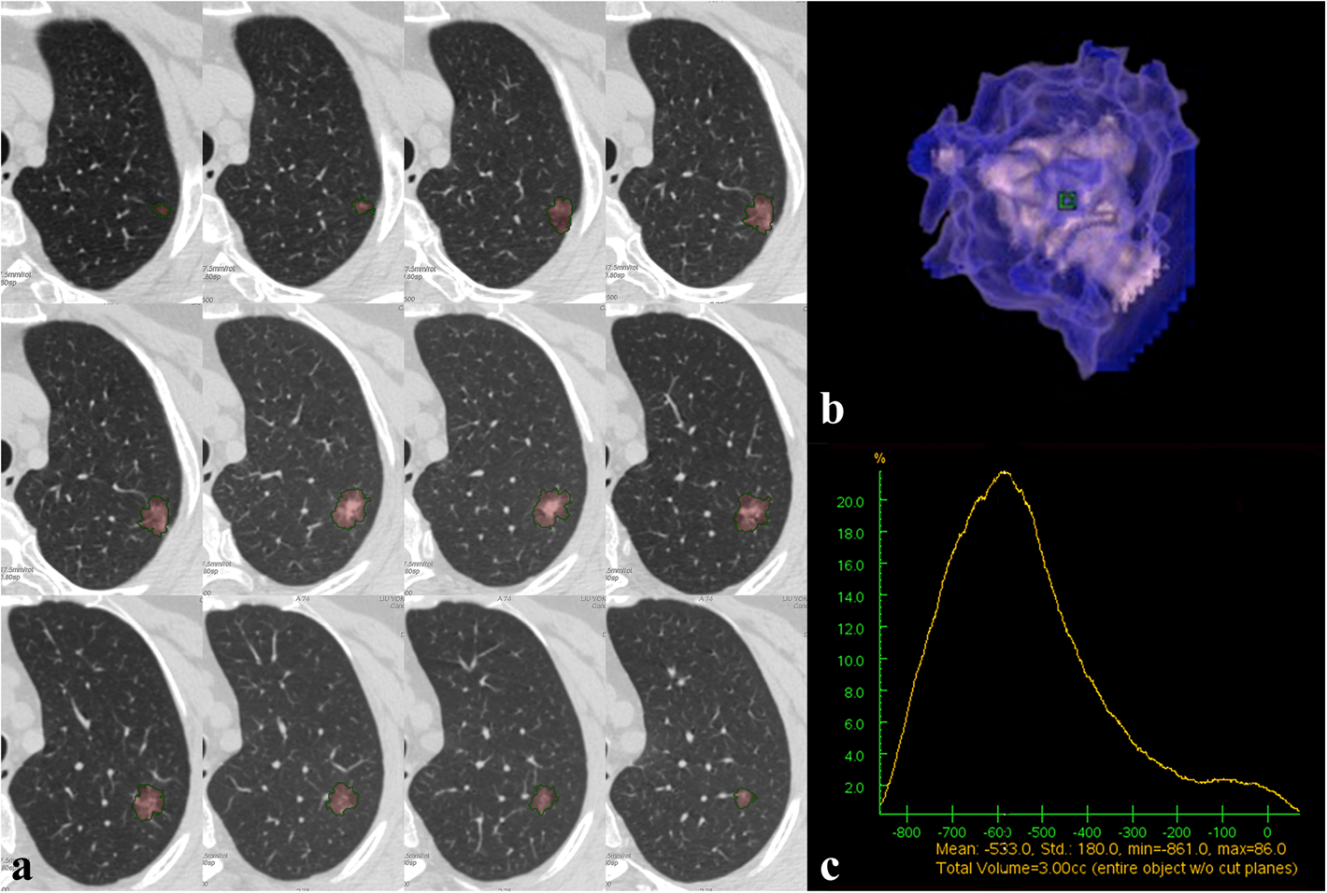
Mean CT value calculated using three-dimensional (3D) computer-quantified CT number analysis. **a** CT images show computer-generated tumor boundaries that were inspected and edited slice-by-slice by radiologist for correctness and consistency. **b** 3D image of the tumor generated automatically by the computer. **c** CT number distribution curve of the tumor. The mean CT value of this tumor is − 533 HU ranging from − 861 HU to 86 HU

**Fig. 2:**
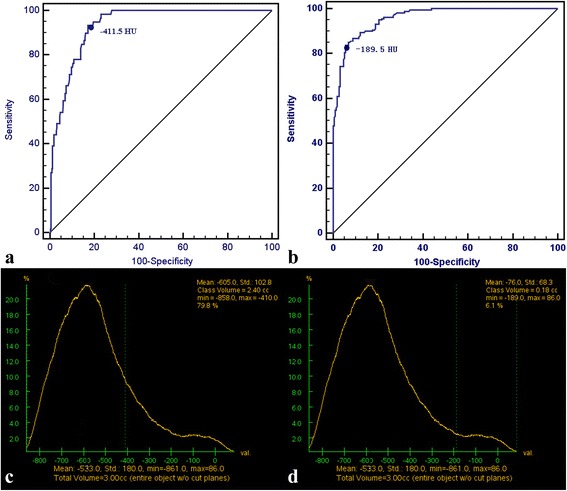
Calculation of the ground-glass opacity component (GGOc) proportion and solid component (Sc) proportion in the CT number distribution curve. **a** The threshold CT values of GGOc were obtained using receiver operating characteristic (ROC) according to the CT appearance. **b** The threshold CT values of Sc were obtained using ROC according to the CT appearance. **c** A case of calculation of the GGOc proportion. The region of CT value≤ − 411.5 HU is defined as the GGOc in the CT number distribution curve, the proportion of which is 79.8%. **d** A case of calculation of the Sc proportion. The region of CT value≥ − 189.5 HU is defined as the solid component in CT number distribution curve, the proportion of which is 6.1%

Three-dimensional computer-quantified CT number analysis was performed on an ADW 4.6 workstation (GE Medical Systems) to measure the proportion of GGOc and Sc. This measurement was performed in three steps as follows. **(a)** Nodule segmentation. The entire tumor mass was separated from surrounding anatomic structures using a computer-aided volume measurement software (Auto Contour in Volume Rendering). Subsequently, a radiologist visually inspected the computer-generated tumor boundaries for correctness and consistency. If any segmentation results were considered suboptimal, the radiologist edited the tumor contours superimposed on the original images. The mean CT value of tumor was automatically tallied up (Fig. 1). **(b)** Aaccording to the CT visual appearance (pGGO, PSN, SN), the threshold values of GGOc and Sc were obtained using receiver operating characteristic (ROC) curves. **(c)** As the second step, the entire tumor mass was separated using the same method in the first step, and the CT number histogram of the tumor was automatically generated by the computer software (3 Dimension Histogram). The proportion of GGOc/Sc was calculated based on the threshold values obtained in the second step (Fig. 2).

To examine the intra- and inter-observer agreement, two radiologists (M.L. and L.Z.) performed the same measurement on 50 randomly selected tumors and the second radiologist (L.Z.) performed the measurement again after one month. The workflow for all the 375 patients was completed by the second radiologist (L.Z.).

### Pathologic evaluation

An experienced lung pathologist with 8 years of experience (W.S.) in lung pathology retrospectively reviewed all resected specimens according to the 2011 IASLC/ATS/ERS classification [3]. For each lesion, histologic subtypes were semiquantitatively recorded in 5% increments. For difficult cases (20 cases), histologic subtypes were assessed through consultation with a lung cancer pathology expert (D.L.). Based on the reported prognosis of lung adenocarcinoma [5–7, 18], AIS and MIA were referred to as non-invasive adenocarcinomas, and other subtypes of tumors were referred to as invasive adenocarcinomas in the present study.

### Statistical analysis

Inter- and intraobserver agreements were assessed using 95% Bland-Altman limits of agreement and the intraclass correlation coefficient. An intraclass correlation coefficient greater than 0.75 represented good agreement. The difference in the CT value for pGGO, PSN, and SN between enhanced and non-enhanced CT imaging was accessed using an independent Student’s *t* test. The relativity between the GGOc/Sc proportion in CT images and the lepidic growth pattern percentage in pathologic specimens was accessed using Spearman’s rank correlation. The prevalence of nominal variables (e.g., tumor appearance, location, contour, intratumoral necrosis and vacuole sign or cavity/cyst in tumor) was compared using Fisher’s exact test. Differences in the mean values between continuous variables (e.g., tumor size, GGOc proportion, Sc proportion and SUV_max_) were compared using an independent Student’s *t* test. Multivariate logistic regression analysis was used to identify the independent factors to predict adenocarcinoma invasiveness. Finally, ROC analysis was performed to evaluate the differentiating performance of logistic regression models in diagnosing invasive adenocarcinoma. All statistical analyses were performed using a commercial software package (SPSS, Inc., an IBM Company, Chicago, IL, USA). A *P* value of less than 0.05 indicated a significant difference.

## Results

### Patient demographics and pathologic evaluation

The clinicopathological characteristics of the 375 patients with lung adenocarcinomas included in the present study are summarized in Table 1. Among all 375 patients, 92 patients (24.5%) had non-invasive adenocarcinomas and 273 patients (75.5%) had invasive adenocarcinomas.

**Table 1:** Patient characteristics

Characteristic	No. of patients (%)
Median age (y)^a^	58.6 ± 9.8 (31 ~ 84)
Sex	
Male	157 (41.9)
Female	218 (58.1)
Smoking status	
Current or former smoker	119 (31.7)
Non-smoker	256 (68.3)
Invasive lobe	
RUL	152 (40.5)
RML	19 (5.1)
RLL	69 (18.4)
LUL	84 (22.4)
LLL	51 (13.6)
Surgical procedure	
Wedge resection	33 (8.8)
Segmentectomy	1 (0.3)
Lobectomy	341 (90.9)
Imaging technique^b^	
Enhanced CT	193 (51.5)
Non-enhanced CT	182 (48.5)
PET/CT	147 (39.2)
Subtype predominance	
AIS	41 (10.9)
MIA	51 (13.6)
Lepidic	49 (13.1)
Acinar	176 (46.9)
Papillary	32 (8.5)
Micropapillary	5 (1.3)
Solid	11 (2.9)
Variants	10 (2.7)
Pathologic Stage ^c^	
IA	173 (47.5)
IB	148 (40.7)
IIA	22 (6.0)
IIIA	21 (5.8)

Note. Unless otherwise indicated, data are numbers, with percentages in parentheses. ^a^ The data are presented as the means ± standard deviation, with ranges in parentheses. ^b^ All patients underwent CT examination. ^c^ Eleven patients did not undergo lymph node dissection; the pathologic stage was made in 364 patients. *AIS*, adenocarcinoma in situ; *MIA*, minimally invasive adenocarcinoma; *LLL*, left lower lobe; *LUL*, left upper lobe; *RLL*, right lower lobe; *RML*, right middle lobe; *RUL*, right upper lobe

### Difference in the mean CT value between enhanced and non-enhanced CT imaging

All patients included in the present study underwent non-enhanced or enhanced chest CT. Among the 375 nodules analysed, 58 nodules showed pGGO, 159 samples were PSN, and 158 samples were SN. No significant difference was detected between non-enhanced and enhanced chest CT groups for pGGO (*t* = − 1.76, *P* = 0.08), PSN (*t* = − 1.72, *P* = 0.09) and SN (*t* = − 0.84, *P* = 0.40) (Table 2.).

**Table 2:** Mean CT value of pGGO, PSN and SN between enhancement and non-enhancement CT scan

Tumor appearance	Number (%)	Mean CT value (HU)	*t*	*P*
pGGO	58	− 553.38 ± 101.1		
Enhancement	33 (56.9)	−533.36 ± 103.79	−1.764	0.083
Non-enhancement	25 (43.1)	− 579.80 ± 92.92		
PSN/mGGO	159	−364.87 ± 129.61		
Enhancement	94 (59.1)	− 379.49 ± 128.80	1.721	0.087
Non-enhancement	65 (40.9)	− 343.74 ± 128.82		
SN	158	−104.24 ± 93.05		
Enhancement	55 (34.8)	−95.67 ± 94.30	−0.845	0.399
Non-enhancement	103 (65.2)	− 108.82 ± 92.51		

*CT*, computer tomogram; *pGGO*, pure ground-glass opacity; *PSN*, part solid nodule; *mGGO*, mixed ground-glass opacity; *SN*, solid nodule; *HU*, hounsfield unit

### Consistency between the GGOc and Sc proportions

The 95% limits of inter- and intra-observer agreements obtained using Bland-Altman analysis to measure the GGOc proportion were − 0.26 to 0.22 and − 0.03 to 0.03, with intraclass correlation coefficients of 0.954 (95% confidence interval [CI]: 0.918, 0.974) and 0.999 (95% *CI*: 0.999, 1.000), respectively (*P* <  0.001). The 95% limits of inter- and intra-observer agreements in the measurement of the Sc proportion were − 0.016 to 0.016 and − 0.03 to 0.04, with intraclass correlation coefficients of 0.999 (95% *CI*: 0.998, 1.000) and 0.998 (95% *CI*: 0.997, 0.999), respectively (*P* <  0.001).

### Threshold values for GGOc and Sc

The optimal threshold value for GGOc was − 411.5 HU (area under the ROC curve [AUC], 0.93; 95% *CI*, 0.91–0.96) with a sensitivity of 93.2% and specificity of 82.9%, and the optimal threshold value for Sc was − 189.5 HU (AUC, 0.96; 95% *CI*, 0.94–0.97) with a sensitivity of 83.6% and specificity of 94%.

### Relationship between the GGOc/Sc proportion and lepidic growth pattern percentage

The proportion of GGOc in the CT images was positively correlated with the lepidic growth pattern percentage in pathologic specimens (*r* = 0.67, *P* <  0.01), while the proportion of Sc was negatively correlated with the lepidic growth pattern percentage (*r* = − 0.74, *P* <  0.01).

### Radiographic features analysis

**Fig. 3:**
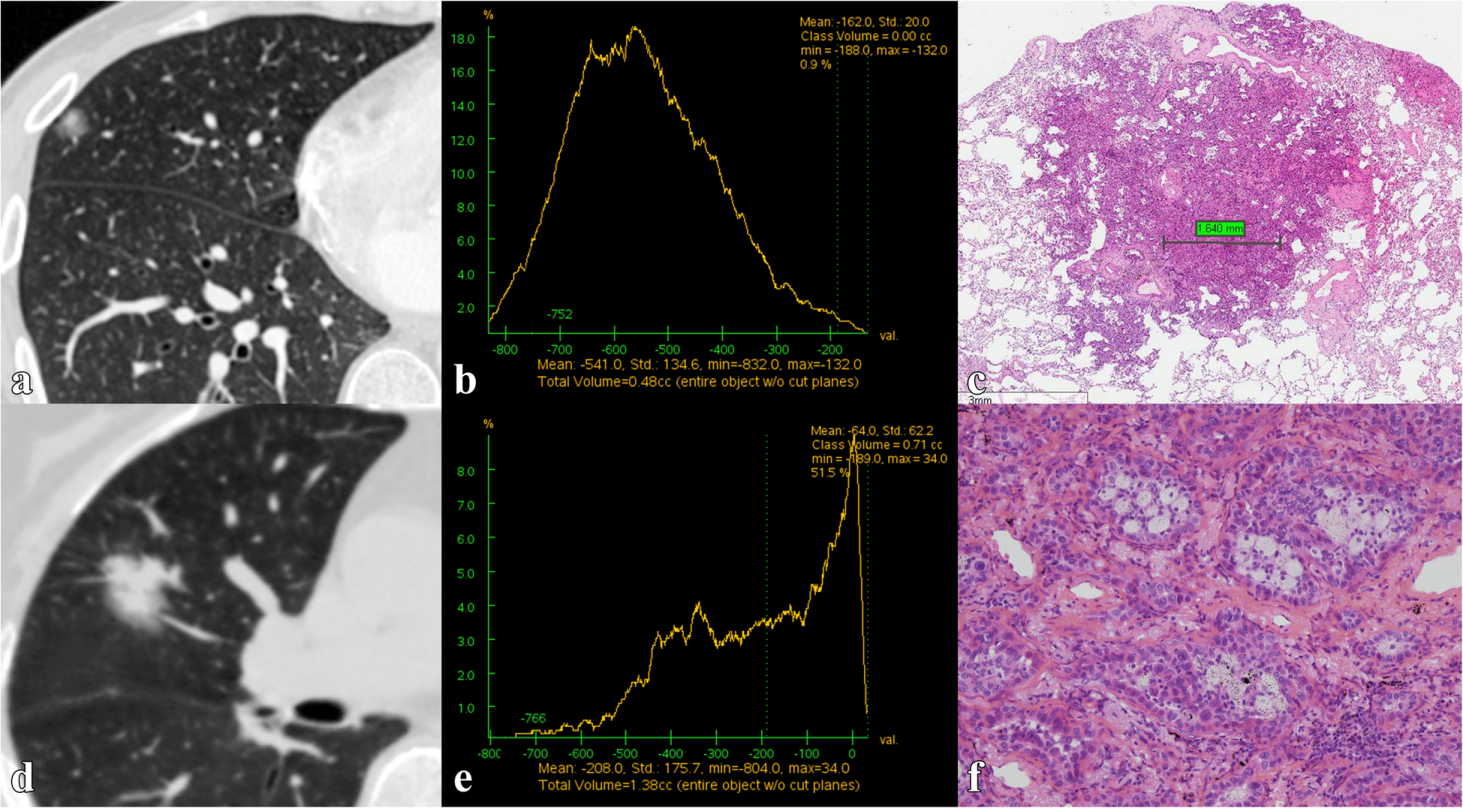
Images in a 46-year-old patient with minimally invasive adenocarcinoma (**a**~**c**) and a 72-year-old patient with invasive adenocarcinoma (**d**~**f**). **a** The lung window of the axial HRCT image shows a part solid nodule in the right middle lobe. **b** The CT number distribution curve presents the solid component proportion of the nodule at 0.9%. **c** Photomicrograph (haematoxylin and eosin stain; magnification × 5) shows minimally invasive adenocarcinoma primarily comprising lepidic growth with a small (1.64 mm) central area of invasion. **d** The lung window of the axial HRCT image shows a solid nodule surrounded by a few ground-glass-opacity nodules in the right middle lobe. **e** The CT number distribution curve presents the solid component proportion of the nodule at 54.5%. **f** Photomicrograph (haematoxylin and eosin stain; magnification × 200) shows acinar predominant invasive adenocarcinoma primarily comprising 70% acinar pattern, 15% lepidic pattern and 15% papillary pattern

Radiographic features of invasive adenocarcinoma and non-invasive adenocarcinoma are presented in Table 3. Tumor size, contour, necrosis, vacuole sign or cyst/cavity, GGOc proportion, Sc proportion (Fig. 3) and SUV_max_ showed significant differences between invasive adenocarcinoma and non-invasive adenocarcinoma (*P* <  0.001, *P* <  0.001, *P* = 0.002, *P* <  0.001, *P* < 0.001, *P* < 0.001, and *P* = 0.018, respectively). Among all 375 patients, only 147 patients underwent PET/CT, thus SUV_max_ was removed from the multivariate analysis. The results of the logistic regression analysis (Table 4) demonstrated that tumor size and Sc proportion were independent predictors of invasive adenocarcinomas (odds ratio [OR] = 2.79, *P* = 0.002, and *OR* = 40.24, *P* < 0.001, respectively).

**Table 3:** Correlation between radiographic features and pathology

Radiographic features	Invasive adenocarcinoma	Non-invasive adenocarcinoma	*χ*^2^/*t*	*P*
HRCT	283 (*n*)	92 (*n*)		
Size^a^	2.04±0.55	1.55 ± 0.51	−7.601	< 0.001
Location			0.295	0.587
Centre	50	14		
Periphery	233	78		
Contour			48.04	< 0.001
Smooth	36	40		
Lobular	143	42		
Spiculated	104	10		
Necrosis			9.415	0.002
Yes	33	1		
No	250	91		
Vacuole sign or cyst/cavity			16.346	< 0.001
No	172	77		
Vacuole sign	89	12		
Cyst/cavity	22	3		
Computer-quantified CT number analysis	283 (*n*)	92 (*n*)		
GGOc proportion^a^	(14 ± 17) %	(44 ± 29) %	9.461	< 0.001
Sc proportion^a^	(56 ± 30) %	(14 ± 21) %	−14.822	< 0.001
PET/CT	137 (*n*)	10 (*n*)		
SUV_max_^a^	3.75 ± 2.85	1.54 ± 2.41	−2.21	0.018

Note. Unless otherwise indicated, the data are the number of patients. a The data are presented as the means ± standard deviation. *HRCT*, high-resolution computer tomography; *GGOc*, glass-ground-opacity component; *Sc*, solid component; *PET*, positron emission tomography; *SUV*, standard uptake value

**Table 4:** Multivariate analysis for invasiveness of lung adenocarcinoma

Variable	Odds Ratio	95% *CI*	*P*
Size (X_1_)	2.79	1.48 ~ 5.27	0.002
Contour (X_2_)	1.11	0.68 ~ 1.82	0.67
Necrosis (X_3_)	0.81	0.09 ~ 7.19	0.85
Vacuole sign or cyst/cavity (X_4_)	1.72	0.96 ~ 3.09	0.07
GGOc proportion (X_5_)	0.17	0.028 ~ 1.002	0.05
Sc proportion (X_6_)	40.24	5.35 ~ 302.73	< 0.001

Note. *CI*, confidence interval; *GGOc*, glass-ground-opacity component; *Sc*, solid component

### Predictive probability of radiographic features for adenocarcinoma invasiveness

**Fig. 4:**
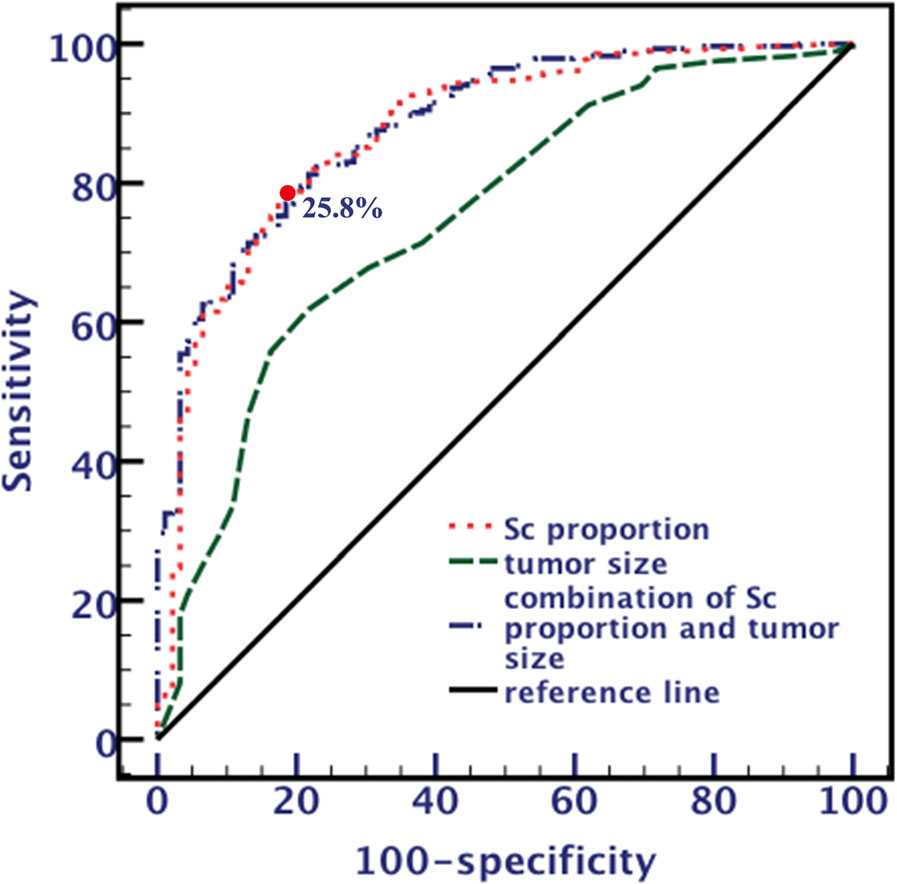
Receiver operating characteristic curve used for analysis of tumor invasiveness with solid component proportion, tumor size and combination of solid component proportion and tumor size. The area under the curve of the combination of the solid component proportion and tumor size is significantly larger than the tumor size but not significantly larger than solid component proportion. The threshold value of the solid component proportion is 25.8%

Based on multivariate analysis, two significant factors were combined (combination of tumor size and Sc proportion) for the prediction of invasive adenocarcinoma, whose AUC value of ROC was 0.884 (95% *CI*: 0.847 ~ 0.922, *P* < 0.001), which was higher than that of tumor size (AUC: 0.750, 95% *CI*: 0.692 ~ 0.808, *P* < 0.001) with a significant difference (*Z* = 4.75, *P* < 0.001). However, no significant difference (*Z* = 1.07, *P* = 0.28) was detected between these combined factors and Sc proportion (AUC: 0.875, 95% *CI*: 0.832 ~ 0.917, *P* < 0.001) (Fig. 4). The optimal threshold for the detection of invasive adenocarcinoma in the Sc proportion was 25.8%, with 78.1% sensitivity and 82.6% specificity.

## Discussions

In the new adenocarcinoma classification, adenocarcinomas with lepidic growth patterns included AIS, MIA, lepidic predominant invasive adenocarcinoma and other adenocarcinomas containing a lepidic growth component. In the lepidic growth pattern, neoplastic cells grow along pre-existing alveolar structures in a flat manner, without forming papillary or micropapillary structures and lacking of stromal, vascular or pleural invasion [3]. Multiple studies have shown that adenocarcinomas with lepidic growth patterns have favourable prognosis and low risk of recurrence [4, 19, 20]. A comparison of high-resolution computer tomography (HRCT) images with pathological sections revealed that areas of GGO in the HRCT image reflect a growth pattern where tumor cells have replaced alveolar lining cells, as in lepidic growth without collapse. In contrast, areas of consolidation primarily represent the foci of fibrosis or tumors of a solid growth pattern, consistent with previous reports [21, 22]. In the present study, the GGOc/Sc proportion measured using 3D computer-quantified CT number analysis was related to the lepidic growth pattern percentage. Tumors with high lepidic growth pattern percentages have low Sc proportion and high GGOc proportion, suggesting that the GGOc/Sc proportion measured using the 3D computer-quantified CT number analysis is accurate and objective and reflects the lepidic growth pattern percentage in pathologic specimens.

To predict invasive adenocarcinoma, we accessed the potential risk factors of radiographic features. We observed that tumor size, contour, necrosis, vacuole sign or cyst/cavity, GGOc proportion, Sc proportion and SUV_max_ were significantly different between invasive adenocarcinoma and non-invasive adenocarcinoma. In the present study, the tumor size of invasive adenocarcinoma is significantly larger than the non-invasive adenocarcinoma, consistent with the current criteria for tumor stage (T stage). We also observed that compared to non-invasive adenocarcinoma, invasive adenocarcinoma is more likely to present as lobular or speculate, necrotic lesion with vacuole sign or cyst/cavity, which demonstrated as malignant signs in lung nodule diagnosis. The GGOc proportion and Sc proportion are quantitative indicators of tumor appearance. In the present study, the GGOc proportion of invasive adenocarcinoma is lower than that of non-invasive adenocarcinoma, while the Sc proportion is higher, consistent with other reports [18, 22, 23]. SUV_max_ is a semi-quantified index of FDG uptake, which is associated with tissue glucose metabolism. Previous studies [24, 25] have reported that SUV_max_ could be affected by cell differentiation and proliferative rate potential. The tumor with high SUV_max_ is more likely an invasive lesion and with an unfavourable prognosis. In the present study, the SUV_max_ of the invasive adenocarcinoma is significantly higher than that of non-invasive adenocarcinoma, which is consistent with previous studies [26, 27].

We analysed these factors, except SUV_max_, using logistic multivariate analysis to assess the joint effects and interactions of the variables on adenocarcinoma invasiveness. These results showed that only tumor size and Sc proportion retained statistical significance, whereas contour, necrosis, vacuole sign or cyst/cavity and GGOc proportion had no significant difference. We proposed that their effects were substituted by the enrolled index. According to the ROC analysis, AUC of Sc proportion was 0.875, which was higher than that of tumor size (0.750, *P* < 0.001) and had no significant difference with that of combination of the two factors (0.884, *P* = 0.28). This result suggested that Sc proportion has higher predicted value than tumor size and has equal efficacy as combined factors. Therefore, we recommend a threshold with an Sc proportion of no less than 25.8% to predict tumor invasiveness, not only because it has favourable efficacy but also because it has good clinical practicability and easy operation.

The major strength of the present study was the measurement of the GGOc/Sc proportion. In the present study, the GGOc and Sc proportions were measured using 3D computer-quantified CT number analysis. Until recently, several measuring methods of the GGOc proportion have been reported. Some researchers [28, 29] calculated the GGOc proportion by measuring the maximum cross-sectional tumor diameter on CT images, which is easy to operate but may only represent a single tumor cross-section instead of the entire tumor mass. In addition, although many studies have calculated the GGOc proportion using computer software for volumetric nodule segmentation, the classic technique has limitations on tumor boundary generation and segmentation on the inner components of tumor [30]. In the present study, the radiologist visually inspected computer-generated tumor boundaries to avoid the vessels and bronchial tubes around the tumor and ensure correctness and consistency; the GGOc and Sc in the tumor were identified using a CT number distribution curve with threshold values obtained from ROC curves, which improved segmentation on the inner composition of the tumor. Intra- and inter-observer agreement assessment demonstrated that 3D computer-quantified CT number analysis is reproducible and reliable in measuring the GGOc/Sc proportion.

In this study, the CT values of SN obtained through the 3D computer-quantified CT number analysis software were much lower than manual measurement in routing work, and there were no difference between non-enhanced and enhanced group. There are several reasons to explain the results. First and foremost, the mean CT value obtained through the 3D computer-quantified CT number analysis was differing from manual measurement. The software measured the entire entire nodule, including the vacuole, cyst/cavity, bronchogram in the tumor, and the surrounding lung tissue, which cannot be excluded absolutely through segmentation automatically by the computer or manually in lung window. The air CT value (− 1000) interrupted and drove down the CT value of SN, and eliminated the influence of contrast injection at the same time. In addition, the partial volume effect in the pixel of the nodule edge will also exert an influence. Second, lung nodules with small quantity of GGO cannot be observed visually and will be classified as solid nodule. However, the small quantity of GGO with extremely low CT number will be accounted by the computer. Last, the delayed scan time is relatively short (25 to 30 s), which had little effect on the attenuation of small lung lesion. This is also why we did not analyse the non-enhanced and enhanced CT imaging separately.

The present study has several limitations. First, the CT examinations in the present study were conducted using different scanners from three companies that may influence the accuracy of the CT value. Second, the present study was conducted as a single-centre study, and additional studies in multiple centres are needed to confirm these results, particularly the threshold values of the GGOc and Sc. Last, we did not conduct survival analyses because the follow-up time for some cases was too short; thus, advanced study is warranted to analyse patient survival.

## Conclusions

The results of this study support the idea that 3D computer-quantified CT number analysis is a reliable method to measure the GGOc/Sc proportion, potentially reflecting the lepidic growth pattern percentage in cT_1_N_0_M_0_ lung adenocarcinoma, and a threshold with an Sc proportion of no less than 25.8% has satisfactory efficacy in tumor invasiveness prediction.

## Abbreviations

ADC: Adenocarcinoma
AIS: Adenocarcinoma in situ
GGOc: Ground-glass opacity component
mGGO: Mixed ground-glass opacity
MIA: Minimally invasive adenocarcinoma
pGGO: Pure ground-glass opacity
PSN: Part solid nodules
Sc: Solid component
SN: Solid nodules
SUV_max_: Maximum standard uptake value
